# Psychosocial work load and stress in the geriatric care

**DOI:** 10.1186/1471-2458-10-428

**Published:** 2010-07-21

**Authors:** Matthias Nübling, Martin Vomstein, Sascha G Schmidt, Sabine Gregersen, Madeleine Dulon, Albert Nienhaus

**Affiliations:** 1 FFAS - Freiburg Research Centre for Occupational and Social Medicine (Freiburger Forschungsstelle Arbeits- und Sozialmedizin), Bertoldstraße 27, 79098 Freiburg, Germany; 2 BGW - Institution for Statutory Accident Insurance and Prevention in the Health and Welfare Services (Berufsgenossenschaft für Gesundheitsdienst und Wohlfahrtspflege), Pappelallee 35/37, 22089 Hamburg, Germany

## Abstract

**Background:**

Due to the decrease in informal care by family members and the demographic development, the importance of professional geriatric care will rise considerably. Aim of this study was to investigate the psychosocial workplace situation for employees in this profession.

**Methods:**

The German version of the COPSOQ (Copenhagen Psychosocial Questionnaire) was used for the assessment of psychosocial factors at work. The instrument includes 22 scales and 3 single items concerning demands, control, stress, support, and strain.

Results between two study groups of geriatric care were compared to each other as well as to employees in general hospital care and a general population mean (COPSOQ database).

Statistical analysis included t-tests, ANOVA and multiple comparisons of means. Statistical significance (p < 0.01, two-tailed) and a difference of at least 5 points in mean values were defined as the relevant threshold.

**Results:**

In total 889 respondents from 36 institutions took part in the study. 412 worked in Home Care (HC), 313 in Geriatric Nursing Homes (GNH), 164 in other professions (e.g. administration).

Comparison between HC and GNH showed more favourable values for the first group for the most scales, e.g. lower quantitative and emotional demands and less work-privacy conflict, better possibilities for development etc. Compared to external values from the German COPSOQ database for general hospital care (N = 1.195) and the total mean across all professions, COPSOQ-total (N = 11.168), the results are again positive for HC workers on most of the scales concerning demands and social support. The only negative finding is the very low amount of social relations at work due to the obligation to work alone most of the time. Employees in GNH rate predictability, quality of leadership and feedback higher when compared to general hospital care and show some further favourable mean values compared to the COPSOQ mean value for all professions. A disadvantage for GNH is the high rating for job insecurity.

A supplementary subgroup analysis showed that the degree of negative evaluation of psychosocial factors concerning demands was related to the amount of working hours per week and the number of on-call duties.

**Conclusions:**

Compared to employees in general hospital care and the COPSOQ overall mean value across all professions, geriatric care employees and especially home care workers evaluate their psychosocial working situation more positive for most aspects. However, this seems partly due to the very high proportion of part-time workers. Critical results for the two study groups are the relatively high job insecurity in nursing homes and the lack of social relations for the HCrs.

## Background

In the years and decades to come, industrial countries will have to face major challenges concerning geriatric care for their population [1]. In the geriatric care sector, the number of people requiring professional care will rise. In recent years, due to the decrease in informal care by family members and the demographic development, there has been a rise in professional care for elderly at home (home care, HC) or in long term care facilities like Geriatric Nursing Homes (GNH) [2]. Based on the statistics of the German Federal Statistical Office, 54% of the 2.25 million people in need of care require professional care in Germany (HC 502.232 and GNH 709.311) [3]. According to current forecasts, an increase of the group of people in need of care of nearly 7% is to be expected until 2010. Totally, the number of persons in need of care will increase to almost 3 million people in 2030 [2], which in turn will cause a rising need of employees in professional care, particularly within the geriatric care sector [4,5]. Simultaneously, a shortage of nursing staff is being expected, due to diminishing willingness to be trained as and to work as a nurse and the lack of financial resources in this health care sector [6].

In summary, for 600.000 trained and untrained nursing staff in HC or GNH [7] this means an increase in workload and work intensity. This especially mental or psychosocial workload - caused by the increase in care intensive clients (HC) and residents (GNH) as well as by the increasingly older nursing staff in the facilities - can lead in turn to growing psychosocial job stress for the nursing staff [8-11].

In the German Health Report 2003 for the long term care sector, nursing staff in GNH reported high time pressure due to high intensity of work [12]. They complained about not having enough time for the support of the residents and that frequently the work had to be interrupted. Further stressors reported were the aggressiveness of some residents, or the handling with incurable diseases and dying. This may have consequences for sickness absence, absenteeism, and for intention to leave the job and job turnover. According to data of a German health insurance company, the average rate of sickness absence of employees in the geriatric care sector was 5.8% (21.3 days/year) compared to an overall rate of 4.9% (17.7 days/year) [13]. Hasselhorn et al. summarize from the results of the European NEXT-Study (Nurses Early Exit Study) that changes in job strain can affect fluctuations and nurses' considerations of leaving their profession [14].

However, research results do not show high strain for the geriatric nursing staff for all aspects. In the German BELUGA-Study (BELUGA = **Bel**astungsanalyse **u**nd **g**esundheitsforderliche **A**rbeitsgestaltung in der Altenpflege; Analyis of stressors and possibilities for health-promoting work design in nursing homes for the elderly) with 1838 participants in 111 geriatric nursing homes and home care facilities the researchers reported for instance a moderate level of stressors in the nursing homes " (e.g. low good cooperation between colleagues and supervisors, which the authors call "low social stressors") and "predominantly positive" indicators of employees' health (irritation, burnout, physical and mental health). Major problems reported were physical work load (lifting), high time-pressure and frequent interruption of work [15].

Altogether for the geriatric care sector the current state of research on psychosocial workload is heterogeneous. Additionally, no literature was found on the systematic comparison between the workload and stress of nursing staff and other occupational groups, which would be necessary to evaluate the workload of the geriatric nursing staff in HC and GNH.

The present study is intended to partly close this gap empirically with a survey on psychosocial workload and stress in geriatric care in the two groups: employees in home care and in geriatric nursing homes (long term care facilities, homes for the aged).

Aims of the present study are to compare the psychosocial factors at work between these two study groups as well as with external reference data for nursing staff in general hospitals and with the general mean across all professions in the COPSOQ database (a database with profession specific reference values collected with the German COPSOQ). A second goal is the analysis of the impact of working schedule aspects (on-call duties, weekly hours worked) on psychosocial factors.

## Methods

### Instrument: the COPSOQ-questionnaire

The well established German version of the COPSOQ (Copenhagen Psychosocial Questionnaire) was used for the assessment of psychosocial factors at work in geriatric care. COPSOQ is a comprehensive tool for the assessment of psychosocial factors at work, originally developed in Denmark by Kristensen and Borg (Danish and English version). The goal of the authors was to develop a questionnaire that was "...theory-based but not attached to one specific theory". The questions and scales are mostly derived from already existing instruments, the content was to be as broad as possible in order to take into account the indeterminateness of the issue of psychosocial factors at work [16,17].

A German standard version was formed in a validation study 2003-2005 with a detailed examination and assessment of the questionnaire's psychometric properties and measuring qualities (e.g. objectivity, validity, reliability of scales, practicability, etc.). Assessment of the reliability, generalisability, construct validity, and criterion validity of the single scales of the COPSOQ showed good measuring qualities for the majority of the scales. Detailed information on the assessment of psychometric properties (in terms of the ISO 10075-3 [18]) and on the content of the questionnaire is given by Nübling et al. [19,20]. Further information is also available on the German COPSOQ website: http://www.copsoq.de (mostly in German).

**Figure 1:**
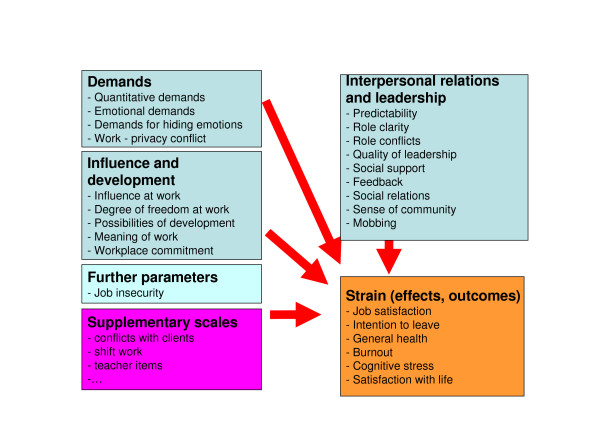
**Content of the German standard COPSOQ**.

The final instrument includes 19 aspects (18 scales and 1 single item) assessing the psychosocial work environment grouped into the sections: demands (4 scales), influence and development (5 scales), interpersonal support and relationship (8 scales and 1 single item), and job insecurity (1 scale) and six constructs (4 scales and 2 single items) assessing the employee's reaction to the workplace situation as outcome factors: job satisfaction, intention to leave, general health, burnout (scale: personal burnout), cognitive stress and satisfaction with life. In total 87 Likert-scaled items with mostly 5 answer categories are included. Figure 1 shows the content of the German standard COPSOQ questionnaire and the direction of the presumed relationship between work-place factors and individual's reactions.

Since 2005 the German COPSOQ standard version has been used for the formation of a comprehensive database with profession specific reference values for psychosocial factors at work. The data of all organizations performing a COPSOQ assessment in cooperation with the FFAS (Freiburger Forschungsstelle Arbeits- und Sozialmedizin; Freiburg Research Centre for Occupational and Social Medicine) were included in the COPSOQ database.

The German COPSOQ database is a dynamically growing data pool which facilitates the interpretation of the results of single organisations/enterprises using comparisons of the results with profession specific reference values.

Professions/occupations in the database are classified according to the system of job classifications KdB92 ("Klassifikation der Berufe 1992") of the German Federal Statistical Office http://www.destatis.de.

In this study some specific questions concerning the working schedule and specific aspects in the care of the elderly (e.g. working with depressed patients) were added to the standard version.

All COPSOQ single items and all scales consisting of several items have a theoretical range from 0 (which is the lowest possible amount of the aspect under investigation) to 100 (corresponding to the highest possible value). Scale values presented are thus mean values and not percentages or prevalence rates, which has the advantage that no information is lost by collapsing categories in order to calculate percentages. Whether a high value is to be judged as positive or as negative depends on the content: a high value for "influence at work" is positive while a high value for "burnout" is negative.

### Performing the Study in Geriatric Care

Invitations to participate in the study were made to n = 75 geriatric care facilities. These were facilities that had indicated to the BGW (Berufsgenossenschaft für Gesundheitsdienst und Wohlfahrtspflege/Institution for Statutory Accident Insurance and Prevention in Health and Welfare Services) in 2007 that they were interested in an employee survey. The BGW offered each of these institutions a free assessment of psychosocial factors at work with the COPSOQ questionnaire, including an evaluation of the facility specific results in comparison to the mean of all geriatric facilities and to reference values from the COPSOQ database.

All in all, 36 geriatric care facilities accepted this offer; these addresses were given to the FFAS, which conducted the survey.

The study was carried out in 2008 by the FFAS as an anonymous survey offered online and in a paper and pencil format. Participation was voluntary, no personal data like addresses were collected - therefore the study did not need to be approved by an ethics committee. Participants sent their filled-out questionnaires in a prestamped anonymous envelope directly to the FFAS; online data were transmitted using a protected internet connection. The FFAS analysed the data and sent reports to every participating institution with comparisons of their results (average of all participants in the institution) compared to the overall mean in all institutions of the same type.

Statistical analysis included descriptive statistics and comparison of mean COPSOQ-scale values using t-tests and Analysis of variance (ANOVA).

Due to the high number of tests (22 scales plus 3 single items, 2 study groups, 2 reference values) and the relatively high number of subjects the significance level was established at p < 0.01 (two-tailed) for comparisons between the two study groups and the COPSOQ reference values. Furthermore, an absolute difference of at least 5 points between the means was postulated as a relevant difference. COPSOQ scales have standard deviations (SD) of about 15-25 points, thus a difference of 5 points corresponds to an effect size of at least 0.2 which is considered as being the threshold of a small effect; for scales with smaller SDs the effect size is than 0.3-0.35 for a 5 point difference. For practicability reasons we decided to stay with the 5 points difference for all scales.

## Results

### Study Sample

Altogether, 36 institutions took part in the survey: 20 of them were of the HC type, 16 were GNH facilities.

The institutions varied greatly in size: the smallest had 15 employees, the largest 350. In total the questionnaire was filled out by 889 employees, corresponding to a response rate of exactly one-third (33.33%), since 2667 employees had been asked to participate. The participation rate varied greatly, however, from one institution to another: the lowest response rate ran to 11%, the highest to 93%.

The subjects were grouped according to their occupation: 412 persons are in the HC group (type of care = "mobile" and profession = "nursing or geriatric care profession" according to the classification of professions, KbB92), 313 subjects form the GNH group (type of care = "stationary" and profession = "nursing or geriatric care profession", again according to KdB92), and 164 of the 889 respondents are not active in nursing or geriatric care occupations but in others occupations inside the geriatric care facilities such as administration, laundry, cleaning etc.

As table 1 shows, the vast majority of those surveyed is female. This is true for the total population of the survey and particularly for the HC group. Furthermore, the older age groups are especially well represented in HC.

**Table 1:** Sociodemographic Characteristics and working schedule of Study Participants

		Total (incl. other professions in the sample); N = 889	HC; N = 412	GNH; N = 313
Gender	Male	98 (11%)	25 (6%)	49 (16%)
	Female	779 (88%)	385 (93%)	260 (83%)
	No answer	12 (1%)	2 (1%)	4 (1%)

Age	- 30 years	112 (13%)	56 (14%)	45 (14%)
	30-39 years	135 (15%)	59 (14%)	61 (19%)
	40-49 years	324 (36%)	180 (44%)	90 (29%)
	50 + years	257 (29%)	103 (25%)	95 (30%)
	No answer	61: (7%)	14 (3%)	22 (7%)

Work hours per week	35+ h/week	383 (43%)	128 (31%)	191 (61%)
	15 - 34 h/week	359 (40%)	196 (48%)	99 (32%)
	<15 h/week	135 (15%)	87 (21%)	19 (6%)
	no answer	12 (1%)	1 (0%)	4 (1%)

On-call duties (n in last month)	None/n.a.	661 (74%)	247 (60%)	263 (84%)
	1-5	172 (19%)	124 (30%)	42 (13%)
	6+	56 (6%)	41 (10%)	8 (3%)

Night shifts (n in last month)	None/n.a.	750 (85%)	372 (90%)	220 (70%)
	1-5	102 (11%)	28 (7%)	69 (22%)
	6+	37 (4%)	12 (3%)	24 (8%)

Shared shifts (n in last month)	None/n.a.	512 (58%)	125 (30%)	234 (74%)
	1-5	281 (32%)	207 (50%)	68 (22%)
	6+	96 (11%)	80 (20%)	11 (4%)

Alternating shifts (n in last month)	None/n.a.	457 (51%)	217 (53%)	110 (35%)
	1-5	254 (29%)	141 (34%)	96 (31%)
	6+	178 (20%)	54 (13%)	107 (34%)

Sum may differ from 100% due to rounding.

Many of the study employees work part-time: 40% of the 889 study participants work 15 to 34 hours per week, and another 15% work fewer than 15 hours per week; in HC, almost 70% of the employees work part-time.

With respect to specific types of shift, the results are differentiated according to the frequency of on-call duties, night shifts, shared shifts and alternating shifts in the past month. One-fourth of the participants served at least one on-call duty in the previous month; in HC the figure is as high as 40%, compared to only 16% in GNH. Around 15% of the respondents worked at least one night shift in the previous month; in HC, at 10%, the proportion is decidedly lower than the geriatric nursing home figure of 30%. 43% of those surveyed put in one or more shared shifts (two shifts or two parts of one shift during a day with free time between) in the previous month; here, again, the HC rate, at 70%, is much higher than the rate for GNH, which only runs to 26%. Almost half of the study population worked at least one alternating shift (rotating schedule between morning, late or night shift); the HC rate of 47% is clearly lower than the geriatric nursing home rate of 65%.

### Psychosocial factors at Work

For all 25 factors in the COPSOQ (19 aspects on the job situation and 6 outcome factors), internal comparisons between the employees in the two study groups GNH and HC and external comparisons to the overall mean of the COPSOQ database (all professions, N = 11168, as of 5/2008), and to the profession specific reference value for general hospital care nurses (N = 1195) were performed.

#### Differences between Home Care and Geriatric Nursing Homes

Table 2 presents the results (means and SDs) for all scales for the two study groups and the profession specific reference values for general hospital care nurses and the overall COPSOQ mean across all professions for all scales. Differences in means are indicated by a "+"-sign, if the study group value is minimally 5 points better than the reference value and by a "-" -sign if the group value is 5 or more points worse than the reference (the significance level of p < 0.01 is regularly reached when differences exceed 5 points). Please note, that the direction of the interpretation varies, "higher" values are not necessarily "better" values - it depends on the content of the scale whether a value implicates a positive or negative result of this aspect of the psychosocial work environment; this evaluation is already done in the "+"- and "-" -signs.

**Table 2:** Study results for HC and GNH and COPSOQ database reference value for general Hospital Care (COPhosp) and COPSOQ database reference value total (all occupations, COPall). Scale means and standard deviations.

Scales and single items	Study: HC N = 412; mean (SD)	Study: GNH N = 313; mean (SD)	HC vs GNH (*)	COPSOQ Hosp. N = 1195; mean (SD)	HC vs COPhosp (*)	GNH vs COPhosp (*)	COPSOQ all N = 11168; mean (SD)	HC vs COPall (*)	GNH vs COPall (*)
**Demands**									

Quantitative demands	46 (21)	56 (19)	HC +	57 (17)	+		59 (18)	+	

Emotional demands	53 (19)	59 (20)	HC +	64 (19)	+	+	58 (20)	+	

Demands for hiding emotions	45 (21)	47 (23)		51 (21)	+		48 (22)		

Work- privacy conflict	42 (29)	48 (30)	HC +	49 (26)	+		53 (29)	+	+

**Influence and development**									

Influence at work	41 (21)	40 (22)		41 (20)			45 (22)		-

Degree of freedom at work	43 (21)	41 (20)		42 (18)			44 (24)		

Possibilities for development	71 (16)	66 (21)	HC +	70 (16)			69 (18)		

Meaning of work	86 (14)	83 (17)		82 (16)			77 (18)	+	+

Workplace commitment	63 (18)	60 (22)		56 (19)	+		56 (20)	+	

**Interpersonal relations and leadership**									

Predictability	66 (21)	61 (22)	HC +	55 (21)	+	+	51 (23)	+	+

Role clarity	80 (15)	79 (17)		78 (15)			74 (18)	+	+

Role conflicts	37 (20)	46 (22)	HC +	47 (20)	+		47 (20)	+	

Quality of leadership	66 (25)	60 (25)	HC +	55 (25)	+	+	47 (26)	+	+

Social support	75 (20)	71 (21)		70 (20)	+		63 (21)	+	+

Feedback	49 (22)	52 (24)		45 (21)		+	39 (23)	+	+

Social relations (quantity)	19 (26)	52 (25)	GNH +	52 (30)	-		45 (28)	-	+

Sense of community	81 (17)	75 (19)	HC +	77 (18)			74 (18)	+	

Mobbing (single item)	14 (21)	22 (25)	HC +	19 (23)	+		18 (23)		

**Additional scales**									

Job insecurity	25 (22)	37 (25)	HC +	33 (22)	+		26 (22)		-

**Strain (effects, outcomes)**									

Intention to leave (single item)	12 (18)	14 (22)		16 (22)			17 (23)	+	

Job satisfaction	68 (15)	64 (17)	sig.	62 (15)	+		62 (16)	+	

General health (single item)	71 (19)	68 (20)		73 (18)		-	73 (18)		-

Personal burnout	42 (19)	45 (20)		45 (17)			43 (19)		

Cognitive stress symptoms	27 (18)	27 (20)		28 (18)			28 (19)		

Satisfaction with life scale	67 (19)	64 (21)		66 (20)			65 (29)		

Differences in means of > = 5 points are indicated by "+": study group value HC or GNH is better than COPSOQ- database reference value for nursery care (COPhosp) or COPSOQ total (COPall) or by a "-": study group value > = 5 points worse than reference value. Remark: all differences reaching or exceeding +-5 points difference are significant with p < 0.01. In the comparison of the two study groups further differences not reaching the 5-point difference but being significant with at least p < 0.01 indicated with a "sig.".

Comparisons of the scale means **between the two study-groups **showed mostly better values for the HC workers (indicated as "HC +"): quantitative demands, emotional demands and work-privacy conflict are lower, possibilities for development and predictability are higher, role conflicts are lower, quality of leadership and sense of community are higher and mobbing as well as job insecurity are rated lower in HC. On the other hand, workers in geriatric nursing homes show higher values for the amount of social relations at work - but this is the only aspect where GNH workers show better values than Home carers (indicated as "GNH +").

#### Differences between HC/GNH and General Hospital Care

Comparisons of the results of the **two study-groups **with the COPSOQ reference value for **general hospital care nurses **(COPhosp) showed for the HC workers that the aspect of having less social relations at work appeared to be the only disadvantage: the amount (the scale assesses amount, not quality) of social relations at work is evaluated as being very low in the HC group. Positive results for the HC employees are lower values on all demands scales, higher workplace commitment, better predictability, fewer role conflicts, better quality of leadership, more social support, less mobbing, less job insecurity and a higher job satisfaction as an outcome factor.

In the geriatric nursing homes we found the following differences compared to the COPSOQ reference value for General Hospital Care: the only negative point is lower self rated general health; positive results from the perspective of the GNH employees are: less emotional demands, better predictability, better quality of leadership, and better feedback quality.

#### Differences between HC/GNH and the COPSOQ overall mean

Comparisons of the **two study groups **with the **COPSOQ overall mean **(mean value of all participants in all professions, COPall) show significant (p < 0.01) and relevant (delta at least 5 points) differences for the following scales (table 2).

In the HC again the only negative finding is the lower quantity of social relations. On the other hand, numerous positive aspects are found (indicated by a plus sign in table 2): the biggest differences of 10 and more points concern lower quantitative demands, less work-privacy conflict, better predictability, less role conflicts, better quality of leadership, more social support, and a better feedback quality.

For the GNH we found fewer significant differences compared to the COPSOQ total. Negative findings were lower influence at work, higher job insecurity, and (as above in comparison to hospital workers) lower self rated general health. Favourable mean values were found primarily in the field of interpersonal relations and leadership, with predictability, quality of leadership and feedback quality showing the biggest differences of at least 10 points.

In total, the psychosocial factors at work are favourable for the HC when compared to each of the other groups: GNH, COPSOQ Hospital Care or COPSOQ overall mean. This is quite obvious for most of the scales in the fields of demands and interpersonal relations and leadership. The only disadvantage for Home Carers is the obligation to work alone most of the time, expressed in the very low value for the scale "social relations".

The situation for GNH is less favourable when compared to HC. However, comparing psychosocial factors at work in this group with the external COPSOQ reference values, positive findings prevail especially in the field of interpersonal relations and leadership. Critical findings are the lower values for influence at work and for self rated health and the high value for job insecurity.

### Subgroup Analysis

The relations between the two working schedule aspects frequency of on-call duties and weekly hours worked and the perception of psychological factors, especially focusing on the demands scales, were investigated in a subgroup analysis.

This analysis was limited to the employees in the two study groups HC and GNH, respondents working in other occupations (administration) were excluded.

#### Subgroup Analysis: on-call duties

**Figure 2:**
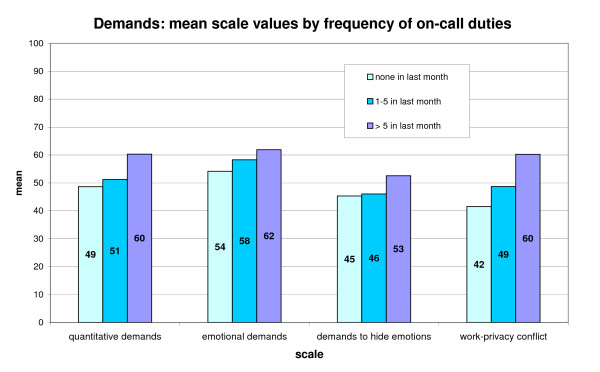
**Demands (4 scales) by frequency of on-call duties**.

In figure 2 the mean values of the four aspects in the field of demands are given for three subgroups formed by the frequency of on-call duties performed in the last month. Most of the 725 study participants in HC and GNH did no on-call duties (N = 510), N = 166 did from 1 to 5 and N = 49 more than 5 on-call duties in the last month.

It turns out that the evaluation of job demands in three of the four demand-scales (quantitative demands, emotional demands, and work-privacy conflict) rises significantly, and more or less linearly (ANOVA, p < 0.01), in proportion to the number of on-call duties performed in the previous month. The differences are particularly clear for the scale work-privacy conflict: here, the mean value for the group with 5 or more on-call shifts is 60 points, compared to 49 points for 1-5 on-call shifts and 42 points for no on-call duty (figure 2).

Further significant differences (all with p < 0.01, data not shown) were found for the aspects: influence at work, possibilities for development, role conflicts, and burnout; all these aspects were higher when performing (more) on-call duties. On the other hand job insecurity and quantity of social relations were higher when not performing on-call duties.

Across all scales the strongest relations found were for quantity of social relations at work and work-privacy conflict.

#### Subgroup Analysis: hours worked per week

**Figure 3:**
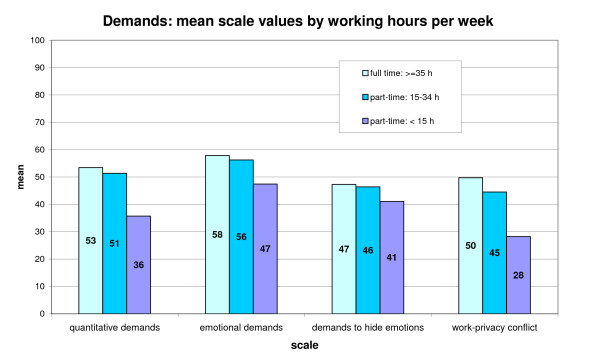
**Demands (4 scales) by working hours per week**.

319 persons in HC and GNH work full time (> = 35 hours), 295 part-time with 15-34 hours per week and 106 employees work less than 15 hours per week (5 with no answer). The analysis of job demands according to the number of hours worked per week (figure 3) shows for three of the four scales a significant (ANOVA, p < 0.01) decrease in reported demands as the weekly hours decrease (a positive effect for those working less hours); for demands to hide emotions the level of significance p < 0.01 was not reached. For the aspects quantitative demands, emotional demands, and work-privacy conflict, however, the decrease is not strictly linear in the three groups. Significantly lower demands appear here only for employees with fewer than 15 hours per week (Scheffé-test: p < 0.01 for all comparisons of this group with both of the other groups for the three scales; no significant differences between the two groups with > = 35 h and 15-34 h).

Other significant differences according to weekly working hours were found for (p < 0.01, data not shown): influence at work, degrees of freedom, possibilities for development (all three in favour for full time workers), role conflicts (higher for full time workers), quality of leadership (higher for part-time employees), quantity of social relations (positive for those working more hours), mobbing (more frequent in full time workers), general health and burnout (both being better for those working only up to 15 hours).

Considering all aspects the relation was strongest for quantitative demands and quantity of social relations.

## Discussion and Conclusion

Psychosocial factors at work were assessed using the theory-based and well established German version of the COPSOQ in 36 institutions in two sectors of geriatric care: HC and GNH.

Psychosocial work load in HC and GNH was compared to reference value of nurses in hospitals in the COPSOQ database and to the COPSOQ overall mean value across all professions.

In a second step the influence of working hours per week and on-call duties on the evaluation of psychosocial factors was analyzed.

One of the main findings was that especially in HC and partly also in GNH the psychosocial workload was evaluated as being lower than in general hospital care by the employees; however this seemed partly to be due to the high rate of part-time workers in HC.

The response rate had a wide range from 11% up to 93% between the 36 institutions in the study - the overall response in the study of 33% is a limitation. From our experience the participation rate is highly dependent on the level of motivation and activation achieved by the local contact persons promoting the survey in the organization - however we have no information about this process for our study. A low response rate might lead to a lack of representativeness and introduce bias into the results.

However, distribution of gender (88% females overall, 93% in HC and 83% in GNH) and the percentage of full time work (43% overall, 31% in HC and 61% in GNH) show that our sample is similar to other findings: Dulon and colleagues [21] found a full time rate of 31% in HC and 53% in nursing homes and a percentage of 90% female workers in HC and 87% in geriatric nursing homes in their study carried out in Germany. The interactive GENESIS-online database of the German Federal Statistical Office https://www-genesis.destatis.de/genesis/online gives for 2009 and for the whole branch of health and social care (no specific data on geriatric care available) a rate of 35% full time workers and 80% female employees.

Regarding the psychosocial factors at work the study produced some results that were expectable, for example the clearly superior rating of the aspect "meaning of work", which is typical for all "helping professions" and has been demonstrated in many other national surveys (e.g. ALLBUS; Allgemeine Bevölkerungsumfrage in den Sozialwissenschaften/German General Social Survey), international surveys (e.g. ISSP, International Social Survey Programme), and studies on hospital workers (e.g. Aust et al. [22], applying the COPSOQ too). The assumption behind this is that the original motivation for the choice of a profession continues to be reflected in the later evaluation of the profession's (positive) characteristics.

Additionally, the study confirmed a phenomenon known from other COPSOQ surveys but up to now not completely explained, namely, the exceptionally positive ratings for "Quality of Leadership" and "Feedback" in the nursing professions.

The ranking in the evaluation of quality of leadership - HC, followed by GNH and Hospital Care - was confirmed in the NEXT study [23] as well.

Theoretically, however, quality of leadership and feedback are not aspects one would expect to see distributed according to occupational group, but according to the specific workplace situation (that is, they are not *job-related*, but *workplace-related*). This often found higher rating in the nursing professions may correspond to the fact that the questionnaire asks for a rating of the *direct *supervisor and that employees tend to experience their direct leaders more as a colleague with some leadership task than a distinct leader (especially since this position is often held by a former colleague).

On a general level, the study also produced some surprising results, for example the rather positive ratings for the "quantitative demands" compared to nurses in general hospital care and the overall mean of all occupations: study results were about average for GNH and markedly below average for HC employees.

Previous studies [12,14,15], on the contrary, point to a high level of quantitative demands, e.g. time pressure, in the nursing professions.

These studies, however, offer no comparison with other occupational groups, which would have made it possible to analyze the reported high level of quantitative demands with reference to external benchmark values.

Several interpretations are possible:

1. Employees in nursing professions might be, on the whole, "less apt to complain", that is, they might "subjectively" report the "objectively" very high demands as being lower than people in other occupations and thus give a value on or below the average, even if the real demands are above. A predisposition or collective reinterpretation of this kind, however, would have to show evidence in the other scales as well, which does not appear to be the case.

2. Over the past decades general changes in working conditions could have led to an overall increase in quantitative demands for all occupational groups - and there are a lot of studies and longitudinal surveys reporting this [24]. In this case, demands would indeed be high in the nursing professions and higher than before, but not conspicuously so.

3. Systematic differences in the forms of employment prevalent among the various professions could lead to systematic differences in the way working conditions are evaluated. Examples of such potential systematic distortions or biases would be part-time employment, temporary employment, precarious employment.

The third hypothesis was tested in the present study in a subgroup analysis. With respondents grouped according to the frequency of on-call duties and according to whether they worked full or part-time, a clear relation emerged between the level of demands they reported on the one hand and the frequency of on-call duties and the number of hours worked per week on the other. The mean scale values rose significantly for three of the four scales in the field of demands according to the number of on-call shifts worked in the previous month. The analysis by weekly working hours, in addition, revealed significantly lower demands for employees not working full time.

The positive results for HC in this study are thus probably due in part to the very high proportion of part-time workers in HC in Germany and not to particularly favourable working conditions (in full time workers less favourable results are found). In our study 21% worked fewer than 15 hours per week and 48% 15-34 hours in HC; in GNH 6% had fewer than 15 hours per week and 32% worked 15-34 hours. And as shown, these high rates of part-time workers are a fact not only in our survey but typical for the German situation.

The results for the scale job insecurity are also interesting. Compared with the mean of all occupational groups, the result for HC is about average. GNH, however, as well as General Hospital Care nurses, appears to be disproportionately concerned with this topic. This may be due primarily to a high level of *qualitative *job insecurity; that is, because of changes in pay scale and contractual conditions, employees in nursing professions may have a heightened fear of not being able to find an *equivalent *position in the event of unemployment or change of job. This would agree with the findings of the NEXT study [25], which showed that in the nursing professions, qualitative job insecurity (e.g. the risk of being transferred to another position) is more of a concern than quantitative job insecurity (the risk of unemployment). In the 4 items of the COPSOQ scale "Job Insecurity" quantitative as well as quantitative aspects are addressed. An analysis on item level reveals the biggest difference for general hospital care when compared to the overall mean in fact for the qualitative aspect of being transferred to another working place without being considered enough: 12 points difference for this item compared to 7 points for the whole scale and 8 points for the worries of becoming unemployed. For the GNH in this study however, the differences are more equilibrated: 10 points for the transfer-item, 14 points for fear of unemployment and 11 points for the whole scale. Thus, fear of unemployment is seen as a problem in both groups but more in GNH than in general hospital care, while qualitative aspects of job insecurity are equally prevalent in both groups and markedly higher than in other professions.

### Strengths and limitations

We are aware of the shortcomings and limitations of mere questionnaire assessments [26]. One point is the general limitation of assessment strategies using one data source only; self-reported data on risk factors and outcomes collected at the same time could lead to the so called "triviality trap" [27,28] or "common method bias"- correlations found could be due to (methodological) artefacts. The linkage of COPSOQ data with other data sources on psychosocial workload would be desirable, for example with data from occupational health physicians or enterprise records or experts' job valuation. This is what Kompier calls the "multi-source" assessment [24]. Some scientific projects are going on in this field, e.g. a survey with police officers where "subjective" COPSOQ data is matched to "objective" data from an in depth check-up by a physician.

But it has also to be said, that only some of the psychosocial factors at work are assessable with validated "objective measures" and that, what is assessed "objectively", is often rather a physical response than an aspect of the psychosocial work situation (rather an outcome than a risk factor). We would therefore agree with Kompier strengthening the job incumbents as experts for their work situation and concluding that the main dichotomy is not "objective" and "subjective" measurement but rather the fact if the criteria of good questionnaire development and appropriate statistical analysis are met in self-report studies.

The COPSOQ-database was established by collecting data from organizations performing surveys together with the research institute - participants may thus not be representative for the working population in general. In ongoing studies applying the German COPSOQ in population-based representative surveys we will be able to control this potential bias.

And - like for the other organisations - a source of potential bias in this survey might be the selection of the HC and GNH facilities based on a pool of facilities that were interesting in an employee survey - again, we do not know if these institutions participating are representative for all geriatric care organisations. On the other hand we found no differences in age and gender for our study population as compared to other studies and the official statistics on the German situation in geriatric care.

We see the major strengths of the formation and use of the COPSOQ-database and of this study in the following points: application of a theory-based, comprehensive and validated survey instrument with a high practicability and usability, and opportunity for comparison of the situation of employees in geriatric care with a large database with profession specific reference data of the last years.

## A remark on adjustment for structural biases

When sociodemographic characteristics or structural conditions (i.e. part-time work) are unevenly distributed between professions and they are related to the work load parameter under analysis too, then an adjustment of the relation between profession and psychosocial factor could be made and is sometimes postulated.

This is true and necessary from a scientific point of view: the comparison between part-time workers (in geriatric care) and full time workers (in other professions) is not "fair", since it is biased by the confounder weekly workload (hours per week). One could argue: "If geriatric care workers worked more, they would demonstrate higher demand values - the low values measured are biased". Or: "If they did no on-call duties, their stress and strain would be much lower". And an adjustment would eliminate the effect of the factor and show more average values.

This is true and correct, but the other perspective is the one of social reality and prevention: what we see in this survey is the reality in the profession of today's geriatric work in Germany: the employees in fact frequently work part-time and on many special schedules, and - importantly - this seems to be a *structural *part of their job (at least as it is organized in Germany). In this case adjustment for working schedules would lead to results which do not exist in reality.

Adjustment or stratified analysis are therefore necessary for the scientific disclosure of the mechanisms behind the self-assessment of psychosocial factors at work (and that is why we did it) - but it may be misleading for the discussion and establishment of preventive or curative strategies in the today's reality of the workplace (and that is why we present the "raw" data).

## Competing interests

The authors declare that they have no competing interests.

## Authors' contributions

Design of the study: AN, SG, MD, and MN. Recruitment of institutions: SG, MD and AN. Organisation of Survey and data collection: MV and MN. Data analysis: MN. Writing of manuscript: MN, MV, SGS, SG, MD and AN. All authors read and approved the final manuscript.

## Pre-publication history

The pre-publication history for this paper can be accessed here:

http://www.biomedcentral.com/1471-2458/10/428/prepub
